# Impact of blended learning on learning outcomes in the public healthcare education course: a review of flipped classroom with team-based learning

**DOI:** 10.1186/s12909-021-02508-y

**Published:** 2021-01-28

**Authors:** Hee Young Kang, Hae Ran Kim

**Affiliations:** Department of Nursing, College of Medicine, Chosun University, 309 Pilmun-daero, Dong-gu, Gwangju, 61452 South Korea

**Keywords:** Curriculum, Flipped classroom, Team-based learning, Public healthcare, Nursing student

## Abstract

**Background:**

A flipped classroom with team-based learning is a blended educational strategy that guides active learning inside and outside the classroom. This study aimed to verify the effects of this innovative blended educational strategy on knowledge, problem-solving ability, and learning satisfaction of undergraduate nursing students undergoing public healthcare education.

**Methods:**

The subjects were undergraduate nursing students enrolled in H University in South Korea. The experiment was conducted over a period of 8 weeks in the public healthcare course. Two groups, blended learning (A flipped classroom with team-based learning) which was the experimental group and traditional lecture-based classroom group, the control group, were assessed. In the blended learning group, the students had pre-class, in-class (including team-based learning elements), and post-class learning elements. The two groups were compared on the following learning outcomes: knowledge, problem-solving ability, and learning satisfaction.

**Results:**

Results showed that the blended learning instructional methods, in comparison with traditional lectures, enhanced the students’ knowledge, problem-solving ability, and learning satisfaction in the public healthcare course.

**Conclusions:**

This study supports the feasibility of the flipped classroom with team-based learning as a blended learning strategy, able to produce improvements in nursing students’ learning outcomes. Blended learning approaches may be an effective alternative to conventional approaches in nursing education.

## Background

In public healthcare systems, nurses play the most significant role and form the major component of most local public health education departments [1]. Public healthcare education, which requires the application of a holistic, multidisciplinary approach and considers the perspectives of various systems, including cooperation among different clinical specialists, is an important strategy to improve national health levels [2, 3]. Therefore, it is important to develop an effective learning strategy for the public healthcare education course in the nursing undergraduate curriculum. The enhancement of healthcare students’ healthcare competencies can improve community healthcare quality and reduce the medical expenses incurred by vulnerable groups [4]. Therefore, expanding the healthcare professionals’ practical experience and collaboration skills to enable them to actively cooperate with professionals from various fields by integrating their knowledge and practical skills, is an important objective of healthcare education courses [5, 6]. Moreover, it is important to improve healthcare education and administration methods to strengthen learners’ self-directed problem-solving and integrated thinking abilities [7]. To overcome the limitations of the traditional lecture (TL) method, the implementation of learner-centered education and active participation of learners in the classroom are necessary [8, 9]. In addition, to overcome the financial constraints of college education and utilize recent developments in educational technology [10], a flexible active learning–based approach should be implemented in healthcare education [11]. Further, the increase in demands for such educational environments has led to the evolution of various teaching models and learning strategies, such as the flipped classroom (FC) approach and team-based learning (TBL).

In the FC, students acquire foundational knowledge through self-directed learning before class and, subsequently, knowledge is transferred from the instructor to the students through instructor-led learner-directed activities. Students then apply this knowledge in the classroom [12, 13]. The FC approach comprises pre-, in-, and post-class learning strategies. In the FC, after the instructor provides various learning contents through an electronic network, students learn them using various digital media, such as their smartphones and notebooks, at their preferred time, space, and speed; subsequently, the students participate in the class [14, 15]. The FC may encourage learners to become independent and creative critical thinkers [16]. However, it seems that the learners perform only limited pre-learning during the pre-class. For example, a study on medical students reported that one-third of students did not participate in the pre-class [17]. Therefore, in the FC, TBL application is likely to promote learners’ active learning. TBL involves the pedagogical use of small groups and intentionally employs specific procedures (e.g., readiness assurance, application activities, and assessment) to transform such groups into active learning teams [18]. This is a teaching strategy that enhances learners’ collaboration ability, disciplinary knowledge, and application ability [19]. Further, TBL has the potential to increase student engagement, satisfaction, and achievement [20]. In particular, the TBL experience helps students with low academic grades to achieve higher grades and improve their attitudes toward the class and pre-class preparation [21]. Therefore, in the FC approach, students are first exposed to learning materials through an online pre-class that they can access wherever they want. In TBL, students interact in small groups and learn together to solve public health care-related problems and reflect on their learnings [22, 23].

The FC with TBL model is based on several pedagogical theories. According to Piaget’s active learning theory, a learner’s interest in collaborative interaction for TBL promotes self-directed learning in the FC [13]. Further, in Bloom’s taxonomy, learners perform low-level cognitive tasks outside the class and high-level cognitive tasks, such as knowledge application, problem analysis, and solution exploration along with their colleagues and instructors inside the class [24]. Therefore, we used TBL within the FC in the undergraduate public healthcare education course.

The FC with TBL approach improves students’ academic achievements by enabling them to learn individually and iteratively, facilitating the sharing of learning content among teams of students, and helping the students achieve high levels of knowledge [25, 26]. Rather than solving tasks outside the classroom, students perform TBL inside the FC. This increases the opportunity for active instructor–learner and learner–learner interactions and, thereby, enhances the students’ problem-solving abilities [27, 28].

Active participation in the learning process also helps improve students’ learning satisfaction [29]. Further, on perceiving the likelihood of positive outcomes, learners stay highly focused on their goals, experience less distress, and achieve higher learning progress. To realize their learning goals, the learners actively participate in the learning process, which includes assessing the demands of assignments, planning relevant strategies, and monitoring the realization of goals [30]. Earlier studies reported that the FC with TBL blended learning approach can more effectively confirm the improvement in learners’ positive recognition than the TL approach [18, 31]. Furthermore, various research results indicated improvements in learners’ academic achievement and learning satisfaction and an increase in the possibility of implementing practical education when learners actively participate in learning content-related activities [32].

Limited research has been conducted so far on the effectiveness of the FC with TBL blended learning approach in the public healthcare education course for nursing undergraduate students. The purpose of this study is to overcome this research gap and compare the effectiveness of the FC with TBL blended learning strategy with that of the TL approach in the public healthcare education course with respect to nursing students’ knowledge, problem-solving ability, and learning satisfaction.

## Methods

### Design and participants

A quasi-experimental design was applied to evaluate the effects of the FC with TBL blended learning strategy on nursing students. To control for any bias arising from contamination, this study considered nursing students who had been in their third year of the public healthcare course in 2014 and 2017 as the TL (control) group and blended (experimental) group, respectively. The subjects consisted of 88 undergraduate nursing students in the 2017 batch and 96 undergraduate nursing students in the 2014 batch enrolled in the H university health department. The participants satisfied the following inclusion criteria: they had no experience in FC and TBL approaches, had no current physical or psychiatric symptoms that could impair their ability to provide informed consent or participate in educational sessions and assessments, and were willing to participate in this study. We assessed the respondents’ psychiatric symptoms using the question “Have you ever felt hopeless or sad for more than two weeks such that it was difficult to live an ordinary life during the last year?” If a participant answered “yes” to the question, we categorized them in the psychiatric symptom group. However, no potential participant replied “yes” in this study. From the 2017 batch, eight transfer students with FC and TBL experience and who provided incomplete questionnaires were excluded. Therefore, the data collected from 90.9% (80) of the participants in the blended learning group and from 93.8% (90) in the TL group were analyzed.

The estimation of the number of samples using G * Power 3.1.4 requires a total of 76 individuals with significance level α = .05, population number = 2, effect size = .50, and power = .95. The effect size was applied to the effect size criterion proposed by Cohen (1992).

### Instruments

To test participants’ knowledge, the research team developed a 23-item multiple-choice questionnaire. Subsequently, two public healthcare nursing professors verified the questionnaire’s content validity. The questionnaire’s total scores ranged from 0 to 30.

This study used the problem-solving ability scale for college students developed by the Korean Educational Development Institute [33]. This scale comprises 45 items to be answered on a five-point Likert scale (ranging from 1 = strongly disagree to 5 = strongly agree). In this study, this scale was used to calculate the average score, and higher scores indicated better problem-solving ability.

Learning satisfaction was measured using the standardized scale of the university’s Teaching and Learning Center (Table 1). Further, this scale was regularly reviewed by the university’s academic advisory committees specialized in teaching and learning. This scale comprises 13 items to be answered on a 4-point Likert scale (ranging from 1 = strongly disagree to 4 = strongly agree). Using this scale, the average score was calculated. Higher scores indicated better learning satisfaction. In this study, the Cronbach’s α value of the scale was 0.88.

**Table 1:** Measurement of participants’ learning satisfaction

Categories	
1	Do you consider the course contents easy to understand?
2	Do you consider the course contents beneficial?
3	Do you consider the course contents interesting?
4	Do you actively participate in class?
5	Do the course lectures proceed systematically as planned?
6	Do you consider the teaching–learning method of the subject matter satisfactory?
7	Do you consider the material used in the course satisfactory?
8	Do you consider the course’s evaluation method appropriate?
9	Do you consider your educational environment (classroom) appropriate?
10	Do you consider your learning environment (outside the classroom) appropriate?
11	Do you consider the implementation of self-directed learning appropriate?
12	Do you consider the equipment required for learning appropriate?
13	Are you satisfied with the details of the course?

### Data collection and procedures

The study was approved by the Human Ethics Committee of H University in South Korea, where the participating students were enrolled. Prior to data analysis, any details identifying the students, such as their names and identification numbers, were replaced with numerical codes.

#### Blended learning design

**Fig 1:**
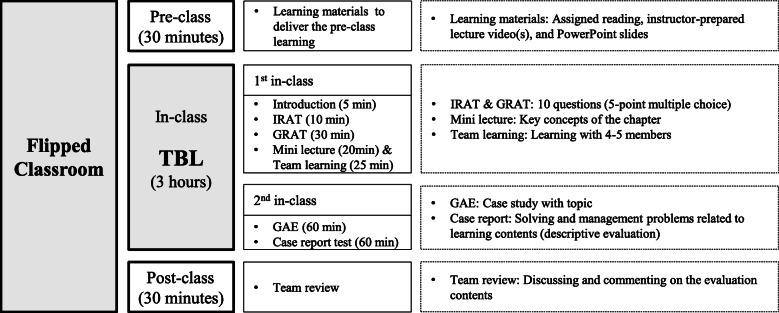
Blended learning design including the FC with TBL approach used in this study. This course was held for eight weeks, including four modules.

Figure 1 depicts the blended learning design, including the FC with TBL approach used in this study. According to this design, four topics were selected as the bases to develop blended learning: public healthcare definition and health policy, understanding international health, epidemiology, and environment and health. These topics were selected because the majority of the questions in the Korean National Examination were from these topics. The module comprised the development of TBL materials for pre- and post-class activities of the FC.

The resources that were made available as pre-class materials included reading assignment contents, instructor-prepared lecture videos, Microsoft PowerPoint slides, and instructor-recorded lectures. Students were given the materials for the class on an online board a week before the class and asked to prepare before attending the class. They were required to learn the pre-class materials for at least 30 min. In class, the students performed TBL, which comprised two phases: individual and group readiness assurance tests (IRAT and GRAT), and a group application exercise (GAE) [18]. During the first in-class session, faculty presented the aim and overview of a lesson (5 min). Subsequently, students took the IRAT (10 min) and GRAT (30 min). The IRAT comprised 10 multiple-choice questions. In the GRAT, each team’s answers were presented on the white board, and each team described how they had arrived at their solutions, as well as the pros and cons that they considered. After the readiness assurance tests, the faculty answered the students’ questions and explained the key concepts of the chapter (20 min). In team learning, students checked the learning objective and included it in the learning plan (25 min). During the second in-class session, students applied the topic’s concepts. They were given materials with a case scenario developed for the GAE (60 min). Subsequently, they discussed and documented the public health problem, and the teams reported their answers during the class itself. After the GAE, the students individually described scenario-related problem-solving and management: the community application method, intervention plans based on evidence, and evaluation methods (60 min). Post-class, students discussed and commented on the topics in a team review. Team reviews were available online, and students had to participate for at least 30 min in a team review. This blended learning continued for 8 weeks, including eight in-class sessions of 2 h each.

#### Traditional lecture

Instead of performing blended learning, students attended TLs and individually analyzed case studies. This was an eight-week course, and each session comprised 2 h. One topic was taught for 2 weeks. During the first session, faculty presented the aim and overview of a lesson (5 min). Further, they explained key concepts, evidence-based interventions, and problem-solving and management methods in the community (100 min). After the lecture, students attended a question-and-answer session (15 min). During the second session, students individually analyzed the case scenario, documented the problem, and performed problem-solving and management of the public health issue (60 min). The case analysis was followed by debriefing (50 min), during which students expressed their thoughts. After the debriefing, students attended a question-and-answer session (10 min).

Before starting the education intervention, students in both groups completed questionnaires assessing their public healthcare education-related knowledge, problem-solving abilities, and learning satisfaction regarding the course that they had previously attended. In the ninth week, students in both groups completed the same questionnaires.

### Statistical analysis

A Chi-square and independent *t*-tests were used to compare the general characteristics. An independent t-test was used to compare the pre-intervention learning outcome variables between the experimental and the control groups. The differences between the two groups in knowledge, problem-solving ability and learning satisfaction in accordance with intervention were analyzed by a one-way repeated measure ANOVA. The collected data were analyzed using the SPSS WIN 24.0 program, as follows: the data were normally distributed, and the verification method was selected. A *p*-values less than 0.05 were considered statistically significant.

## Results

### General characteristics of participants between the two groups

Table 2 offers the general characteristics of the participants. Overall, more than 80% of the participants were women. The mean age of the experimental group and control group was 22.45 and 22.40, respectively. No significant differences were found between the two groups in gender, satisfaction with nursing, and grade point average. Grade point average is the official grade given from the first year to second year and has an available range of 0–4.5.

**Table 2:** General characteristics of participants between the two groups

Characteristics	Categories	Blended Learning (*n* = 80)	Traditional Lecture (*n* = 90)	*χ2* or *t*	*p*
Age: Mean (SD)		22.45 (0.97)	22.40 (0.87)	0.33	.737
Gender: n (%)	Female	65 (81.3)	78 (86.7)	0.93	.335
	Male	15 (18.7)	12 (13.3)		
Satisfaction with nursing: n (%)	Satisfied	16 (20.0)	16 (17.8)	0.19	.908
	Moderately satisfied	59 (73.8)	69 (76.7)		
	Unsatisfied	5 (6.3)	5 (5.6)		
Grade point average: Mean (SD)		3.56 (0.39)	3.62 (0.46)	−0.95	.343

Blended Learning: flipped classroom with team-based learning; *SD* Standard deviation

### Differences in learning outcome variables between the two groups

No significant differences were found between the two groups in pre-intervention learning outcome variables. The blended learning significantly improved all the learning outcomes scores. Using repeated measures ANOVA, the knowledge score showed statistically significant differences in the interactions between the groups (F = 4.48, *p* = .036), between the measurement points groups (F = 464.30, *p* < .001), and between the groups and the measurement points groups (F = 14.45, *p* < .001). The problem-solving ability score showed statistically significant differences in the interactions between the groups (F = 185.04, *p* < .001), between the measurement points groups (F = 783.56, *p* < .001), and between the groups and the measurement points groups (F = 322.69, *p* < .001). The learning satisfaction score showed statistically significant differences in the interactions between the groups (F = 33.41, *p* < .001), between the measurement points groups (F = 311.78, *p* < .001), and between the groups and the measurement points groups (F = 36.34, *p* < .001) (Table 3).

**Table 3:** Differences in learning outcome variables between the two groups

Variables		Blended learning	Traditional lecture	*p* ^a^	Source	F	*P* ^b^
		Mean (SD)	Mean (SD)				
Knowledge	Pretest	18.63 (3.76)	19.57 (3.72)	.103	Group	4.48	.036
	Posttest	25.45 (2.56)	24.34 (3.42)		Time	464.30	<.001
					GroupxTime	14.45	<.001
Problem-solving ability	Pretest	3.41 (0.24)	3.43 (0.23)	.638	Group	185.04	<.001
	Posttest	4.54 (0.30)	3.67 (0.26)		Time	783.56	<.001
					GroupxTime	322.69	<.001
Learning satisfaction	Pretest	2.69 (0.39)	2.68 (0.35)	.910	Group	33.41	<.001
	Posttest	3.98 (0.53)	3.32 (0.72)		Time	311.78	<.001
					GroupxTime	36.34	<.001

Blended learning: flipped classroom with team-based learning; *SD* Standard deviation. ^a^ Score from the independent t-test. ^b^ Score from the repeated measures ANOVA

## Discussion

The results of this study expand earlier findings on how blended learning, including the FC with TBL model, can enhance learning outcomes in education [31]. Moreover, this model was found to increase the effectiveness of teaching and learning methods to improve knowledge acquisition, which is consistent with the results of several other studies [34, 35]. This unique three-process blended learning model is much more helpful in expanding participants’ knowledge than traditional classrooms. Further, the use of various pre-learning materials can help learners engage in self-directed learning [14]. It is noted that the FC does not merely involve the acquisition of knowledge outside the classroom. Rather, it involves pre-class preparation for team learning as the first step in nurturing active learners, who can perform high-level cognitive work. In addition, for successful blended learning, the composition of activities outside and inside the class must be consistent [36]. In an FC with TBL, educators should carefully consider course design and develop consistent learning flows from pre-class online content to TBL content in class and content for post-class reflection. This helps students participate in effective learning activities and maintain positive attitudes toward learning outcomes [37]. In addition, within the classroom, learners can experience peer instruction through TBL. Following the class, learners recognize their role as team members and expand their knowledge through a continuous self-assessment process.

In particular, the FC with TBL model provides learners with pre-class videos related to the topic. Since such videos can be easily accessed using smartphones and notebooks, they enable self-directed learning that transcends time and space. Further, participants familiar with smart devices do not encounter any difficulty in accessing these videos [38]. Hence, smart devices enable individual and iterative learning and facilitate team interactions. This seems to be an environmental factor that facilitates students’ active learning.

Further, the FC with TBL model was effective in enhancing participants’ problem-solving abilities by facilitating case studies requiring teamwork. To provide public healthcare, healthcare professionals must collaborate with experts. Finding and solving real-world public healthcare problems is a difficult endeavor for an individual healthcare professional. Hence, the main goal of TBL is to enable team development and team-centered problem solving [39]. Members of the FC with TBL group regularly demonstrated high levels of learning by applying, analyzing, evaluating, and even acquiring information during team activities depending on their understanding of public health [40]. Therefore, the current study revealed that participants’ problem-solving ability improved in the blended learning group.

The blended learning group members reported more positive learning satisfaction than the TL group members. The FC with TBL model is a student-centered approach and, hence, is different from traditional classrooms, which are mostly instructor centered and provide one-sided learning materials. Earlier studies suggest that learner-centered discussions and teacher facilitation behaviors expressed through learning activities help participants feel valued in a learning environment [41, 42]. Further, participants can break the general boundaries of classroom instruction and achieve their learning goals by themselves both inside and outside the classroom. In particular, the predelivery of video lectures, which is a component of pre-class, enables participants to have a flexible framework that lets them decide for themselves when, where, and how to study in accordance with their personal learning pattern. This enables learners to perform self-directed learning, use familiar tools for learning, and maximize their positive awareness of the FC with TBL approach.

To implement such blended learning, it is important that information is transferred from resources such as videos to learners during pre-class learning [12]. Since the pre-class operation involved only limited student access through the university’s online system, this study delivered the required materials through social networking platforms and cloud technology. Videos were made using advanced technologies to ensure that learners could study without feeling bored. This further ensured that participants could access the learning materials relatively easily and create a deliberate but self-directed and autonomous learning environment. In this environment, learners could study at their own pace, which considerably improved their learning outcomes.

Very few studies in Korean nursing education use the FC with TBL model as a blended learning strategy to encourage students’ active participation. In this study, we applied this educational strategy to a public healthcare education course to promote outside-the-class learning associated with the FC, active learning, team interactions, and reflection. Our findings suggest that FC with TBL is a suitable model to teach public healthcare education courses, as evidenced by the improvements in learning outcomes. Finally, in this study, the FC with TBL model was evaluated in broader terms, such as its ability to increase problem-solving ability and learning satisfaction, over providing simple knowledge by standardized tools.

### Limitations

This study has some limitations. The blended learning method discussed in this study can be improved with respect to the time, costs, and materials required for planning and content preparation, including videos for pre-class learning. Further, there is a two-year interval between the experimental and control groups; however, it is reasonable to believe that this delay did not affect the study results because the participants’ curriculum, educational environment, and research conditions remained the same over the years [17]. The participants in the two groups had different admission years and were asked to maintain the contents and evaluations of the class confidential; however, we did not check for contamination between the two groups. In this study, participants were third-year nursing students, and the scenario was limited to a public healthcare course. Hence, caution must be exercised when generalizing the results to applications with other levels of nursing students or to other nonpublic health content. We recommend that future studies on blended learning strategies include qualitative and observational components to more clearly ascertain a broader array of behavioral, cognitive, and motivational outcomes and, perhaps, elucidate the mechanisms by which FC and TBL affect student learning. Further, this study does not provide adequate data for long-term information retention. Finally, the psychiatry symptom variable was measured subjectively using simple questions, rather than standardized tools. Therefore, to obtain objective data on these variables, future studies should use standardized assessment scales.

## Conclusions

This study found that the FC with TBL blended learning model enhanced the knowledge, problem-solving ability, and learning satisfaction of third-year Korean nursing students. Furthermore, it promoted self-directed learning. In addition, this educational strategy was found to be effective both outside and inside the classroom to obtain positive learning outcomes. Finally, we suggest that the FC with TBL approach is one of the most suitable methods for teaching public healthcare courses and may enhance nursing students’ cognitive abilities at a higher level.

## Data Availability

Not applicable.

## Abbreviations

FC: Flipped classroom
GAE: Group application exercise
GRAT: Group readiness assurance test
IRAT: Individual readiness assurance test
TBL: Team-based learning
TL: Traditional lecture
